# Damaged Keratin Filament Network Caused by *KRT5* Mutations in Localized Recessive Epidermolysis Bullosa Simplex

**DOI:** 10.3389/fgene.2021.736610

**Published:** 2021-11-29

**Authors:** Fuying Chen, Lei Yao, Xue Zhang, Yan Gu, Hong Yu, Zhirong Yao, Jia Zhang, Ming Li

**Affiliations:** ^1^ Department of Dermatology, Xinhua Hospital, Shanghai Jiaotong University School of Medicine, Shanghai, China; ^2^ Institute of Dermatology, Shanghai Jiaotong University School of Medicine, Shanghai, China; ^3^ Experiment Medicine Center, The Affiliated Hospital of Southwest Medical University, Luzhou, China; ^4^ Academician (Expert) Workstation of Sichuan Province, The Affiliated Hospital of Southwest Medical University, Luzhou, China

**Keywords:** recessive EBS, KRT5, genodermatosis, desmoglein 1, keratin

## Abstract

Epidermolysis bullosa simplex (EBS) is a blistering dermatosis that is mostly caused by dominant mutations in *KRT5* and *KRT14*. In this study, we investigated one patient with localized recessive EBS caused by novel homozygous c.1474T > C mutations in *KRT5*. Biochemical experiments showed a mutation-induced alteration in the keratin 5 structure, intraepidermal blisters, and collapsed keratin intermediate filaments, but no quantitative change at the protein levels and interaction between keratin 5 and keratin 14. Moreover, we found that MAPK signaling was inhibited, while desmosomal protein desmoglein 1 (DSG1) was upregulated upon *KRT5* mutation. Inhibition of EGFR phosphorylation upregulated DSG1 levels in an *in vitro* model. Collectively, our findings suggest that this mutation leads to localized recessive EBS and that keratin 5 is involved in maintaining DSG1 *via* activating MAPK signaling.

## Introduction

Intermediate filaments (IFs) protect the epidermis against mechanical and other stresses and play an important role in physically reinforcing keratinocytes and contributing to strong intercellular adhesion *via* their interactions with hemidesmosomes and desmosomes (Kim and Coulombe, 2007; Homberg and Magin, 2014; Chen et al., 2020). IFs are obligatory heteropolymers of type I and type II keratin polypeptides, among which keratin 14 (K14) is the obligatory binding partner of keratin 5 (K5). Dominant mutations in keratin 5 (*KRT5*) or keratin 14 (*KRT14)* lead to epidermolysis bullosa simplex (EBS), which is characterized by intraepidermal blisters, tissue fragility, and collapse of the keratin network into cytoplasmic protein granules, especially upon mechanical stress and other types of stress (Kellner and Coulombe 2009). Keratin aggregates reflect the accumulation of misfolded proteins (Hatzfeld and Burba 1994). Approximately 5% of all *KRT14* mutations that cause EBS have been reported to be recessive, and Yasukawa et al. reported recessive *KRT5* mutation in dominant and recessive forms of EBS (Yasukawa et al., 2002). The aim of this study was to study the effects of homozygous c.1474T > C mutations in *KRT5* on the pathogenesis of recessive EBS.

## Materials and Methods

### Case Report

**FIGURE 1:**
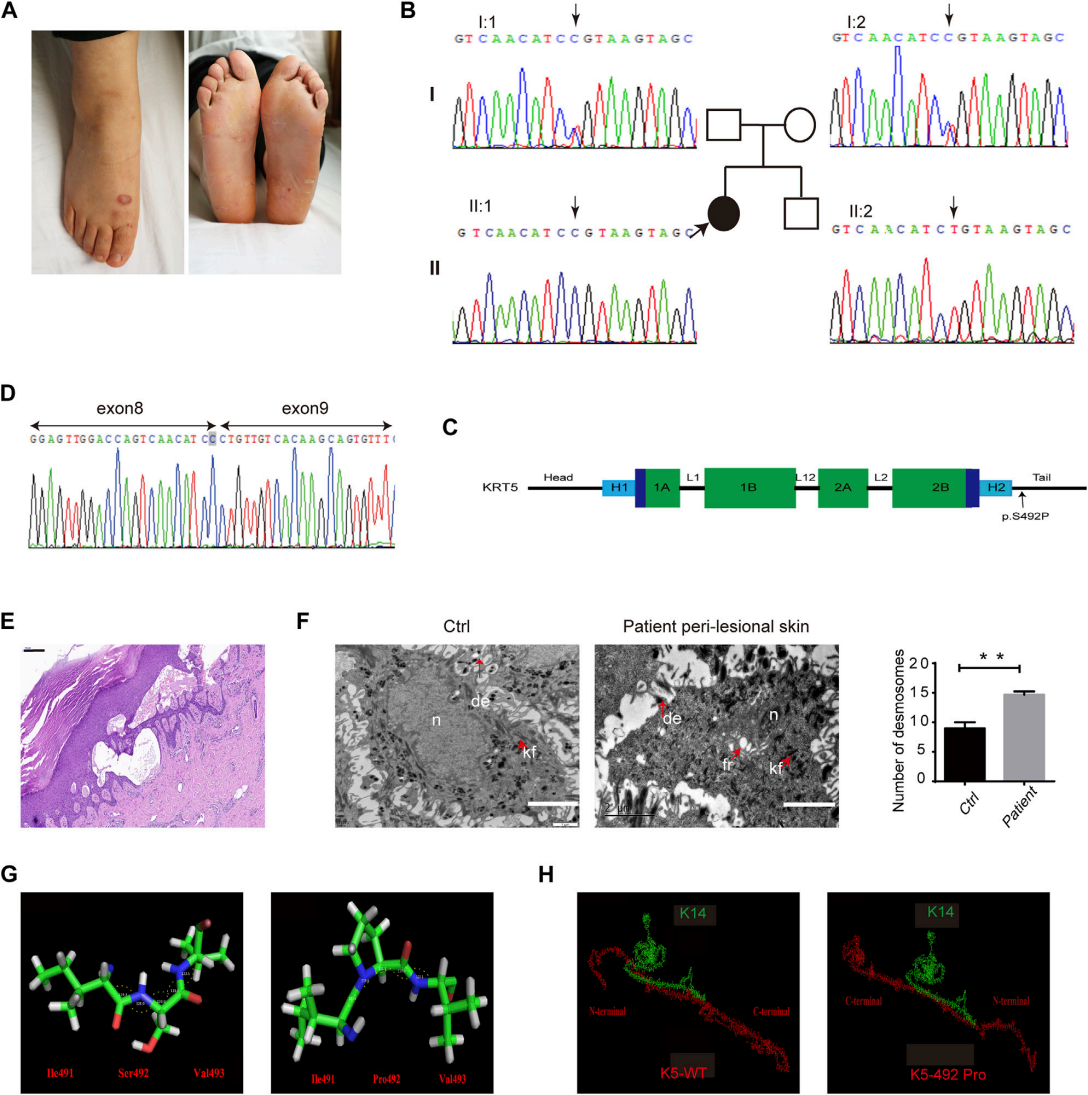
Clinical, molecular, and histological consequences of the *KRT5* mutation. **(A)** The patient showed localized blistering. **(B)** Partial *KRT5* sequence from the patient (II:1) shows the homozygous missense variant c.1474T > C, which was inherited from both of her unaffected parents. **(C)** Schema of the protein domains of K5 and the location of the mutation in the proband. **(D)** RNA analysis indicated that the mutation did not influence mRNA transcription but changed a serine residue to a proline residue. **(E)** Affected skin patches showed extensive epidermal thickness and intraepidermal blisters. Bar, 200 µm. **(F)** Transmission electron microscopy (TEM) showed multiple fractures in the cytoplasm, enlarged intercellular spaces, the clumping of keratin filaments, and increased numbers of desmosomes **(right)** compared to the normal control **(left)**. kf, keratin filaments. de, desmosomes. fr, fracture. n, nucleus. Bars, 2 µm. Statistical significance was assessed by unpaired two-tailed Student’s *t*-test. *n* = 3, **p < 0.01. **(G)** The structures of three residues and angles between their atoms (Ile491-Ser492-Val493) in wild-type and in mutant K5. These angles were 114.3°, 128.0°, 109.0°, 118.0°, and 123.5° in wild-type K5 and 123.5°, 119.6°, 111.1°, 117.0°, and 122.1° in mutant K5, respectively. **(H)** The interactions between wild-type K5 and mutant K5 with K14.

In this study, we investigated a 29-year-old woman, who developed mild blisters with a lifelong history in regions exposed to natural mechanical strain, namely, the chest, back, groin, and feet. No pigment abnormalities, figurate hypopigmentation, or incipient palmoplantar keratoderma were noted (Figure 1A). In addition, we could not detect nail changes, and the oral mucosa was not affected. The skin lesions gradually relieved with age in proband. Her family history was negative.

### Ethics Approval

This study was approved by the Ethics Committee of Shanghai Jiaotong University School of Medicine and conducted in accordance with the principles of the Declaration of Helsinki.

### Mutation Identification and RT-PCR

We sequenced the exomes of the proband *via* whole exome sequencing. The targeted exon was enriched and sequenced on an Illumina HiSeq2000 platform (Illumina). Sanger sequencing was subsequently performed on proband, her asymptomatic relatives (parents and brother), and 100 unrelated healthy Chinese controls to verify the likely pathogenic variants (Li et al., 2013).

Total RNA from patient hair follicles was extracted using Trizol reagent (TAKARA) according to the manufacturer’s instructions. We used TransScript First-Strand cDNA Synthesis SuperMix (Transgen Biotech) to amplify cDNA from 500 ng of total RNA. Mutation was identified by comparing with the reported cDNA reference sequence (NM_000424.3). A 223-bp fragment spanning exon 8 of the *KRT5* gene was amplified by PCR using the forward primer: AGT​ATG​AGG​AGA​TTG​CCA​AC, reverse primer: AGT​AGT​AGC​TTC​CAC​TGC​TA. Sequence comparisons and analysis were performed using the Phred-Phrap-Consed program version 12.0 (http://www.phrap.org/phredphrapconsed.html).

### *KRT5* Constructs

A complementary DNA clone consisting of the entire open reading frame of *KRT5* was cloned into the pCXN2.1 vector with the *Hind*III and *Bam*HI enzymes. The c.1474T > C mutation was introduced into the pCXN2.1-*KRT5* vector using the QuikChange site-directed mutagenesis kit (Stratagene, La Jolla, CA) and mismatched primers (Niwa et al., 1991). Primers used for site-directed mutagenesis were manually designed based on *KRT5* sequence information. Mutations were verified by sequencing using ABI Big Dye terminator reaction mixture (Life Technologies, Carlsbad, CA) according to the manufacturer’s recommendations.

### Cell Culture and Transfection

Human HaCaT cells–human normal skin immortalized keratinocyte strain (from Cell Bank of Type Culture Collection Committee of Chinese Academy of Sciences) were cultured in DMEM medium supplemented with 10% fetal bovine serum (Invitrogen, Switzerland), 1% penicillin, and 1% streptomycin in a 35-mm Petri dish at 37°C and 5% CO_2_. Cells were transiently transfected with 2.5 g wild-type (WT) or mutant pCXN2.1-*KRT5* vector using Lipofectamine 3000 (Invitrogen) (5 ul P3000™ and 7.5 ul Lipofectamine™ 3000) per dish for 48 h according to the manufacturer’s instructions. Twenty-four, 48, 72, and 96 h after transfection, we performed RNA analysis and immunoblot analysis to verify transfection efficiency. Then, we performed immunoblot analysis and immunostaining analysis to investigate the changes upon *KRT5* mutations in HaCaT cells.

### Immunoblotting

Skin biopsy and cell samples were lysated with RIPA buffer, separated in 10% SDA-PAGE gels, and transferred to PVDF membranes (ThermoFisher Scientific; 88518). The membranes were blocked in 2% BSA for 1 h at room temperature and incubated with the primary antibodies (Supplementary Table S1) overnight at 4°C. Then, they were incubated with the secondary antibody for 1 h at room temperature and then detected with SuperSignal West Femto Maximum Sensitivity Substrate (Thermo Fisher Scientific; 34095).

### Co-Immunoprecipitation (Co-IP) Analysis

Proteins prepared from skin and HaCaT cells were collected in Co-IP lysis buffer with 2.5% cocktail and 1% PMSF. Proteins of interest were enriched using anti-K5 antibody (diluted 1:100) immobilized on Protein G agarose beads. The enriched proteins were analyzed by SDS-PAGE (Supplementary Table S1).

### Cell Inhibition Assay

Cells were maintained in growth medium overnight prior to drug treatment. Then, erlotinib HCl (Selleck, United States) was added as a single agent at various concentrations (0, 0.15, and 0.25 µM for HaCaT cells and 0, 4, and 8 µM for HEK-293T cells) to inhibit EGFR signaling, followed by incubation for 96 h.

### Confocal Immunofluorescence Microscopy

Samples were fixed with 4% paraformaldehyde, permeabilized, and blocked in Immunol staining blocking buffer (Beyotime, China) for 1 h at room temperature. Samples were incubated with primary antibodies at 4°C. The samples were then incubated with secondary antibody for 1 h and DAPI for 5 min. A Leica DMIRE2 digital scanning confocal microscope (Leica Microsystems, Germany) was used to detect fluorescence and obtain images. The filters used were appropriate for the detection of Alexa Fluor 488, Alexa Fluor 568, and Alexa Fluor 405. Images were processed using ImageJ, Photoshop CS5, and Adobe illustrator software.

### Statistical Analyses

Statistical analyses were done using GraphPad Prism 6.0 software (La Jolla, California, United States). Results are reported as mean ± SEM. Statistical significance was assessed by unpaired two-tailed Student’s *t*-test. When an experiment contained three groups of values, statistical significance was assessed by ordinary one-way ANOVA, followed by Bonferroni’s multiple comparison. Differences were considered significant when *p* < 0.05.

## Results

### *KRT5* Mutations in Autosomal Recessive EBS

Identified homozygous mutations: c.1474T > C (p.Ser492Pro) in the tail domain of *KRT5*, located in the last amino acid in exon 8, which may affect splicing (Figures 1B,C). Sequencing analyses revealed that both biological parents were heterozygous carriers of the *KRT5* gene mutation, whereas her healthy brother was negative for both variants (Figure 1B). This mutation converts Ser492 to Pro492 and was found to co-segregate in this Chinese family; the mutation was not detected in 100 unrelated healthy Chinese individuals (200 alleles) by Sanger sequencing. We then performed RNA analysis of the patient’s hair follicles, which indicated that the c.1474T > C mutation changed a serine residue to a proline residue and did not influence splicing (Figure 1D).

Affected skin patches showed a thicker epidermis and intraepidermal blisters (Figure 1E). Ultrastructural analysis showed splits within the basal keratinocytes, enlarged intercellular spaces, and the clumping of keratin filaments mainly in basal layer, and increased desmosomes in upper epidermis (Figure 1F; Supplementary Figure S1). However, no obvious abnormalities were observed in non-lesional patient biopsy.

### Bioinformatics Analysis to K5

PolyPhen-2, Mutation Taster, ACMG, and the CADD server predicted that this mutation most likely has a deleterious effect on K5 structure. Protein modeling indicated that the Ser492 residue is located at the linker between two α-helices. With a proline [nonpolar amino acid, side chain: (CH_3_)_2_-CH-CH_2_-] residue instead of a serine [polar amino acid, side chain: HN = C(NH_2_)-NH-(CH_2_)_3_-] residue in this position, an obvious change in the bond angle between the 492nd residue and the adjacent residues occurred (Figure 1G). This type of change in the protein skeleton may significantly affect its function. Analysis of the interaction between K5 and K14 indicated that the p.Ser492Pro mutation may significantly disturb docking of the two proteins (Figure 1H) and that the interface residues were reassembled (Supplementary 1). Furthermore, the energy of this protein complex changed from −109.06 (WT) to −87.25 (mutant).

### Mutant K5 Maintains Protein Stability but Causes Collapse of the IFs Network

**FIGURE 2:**
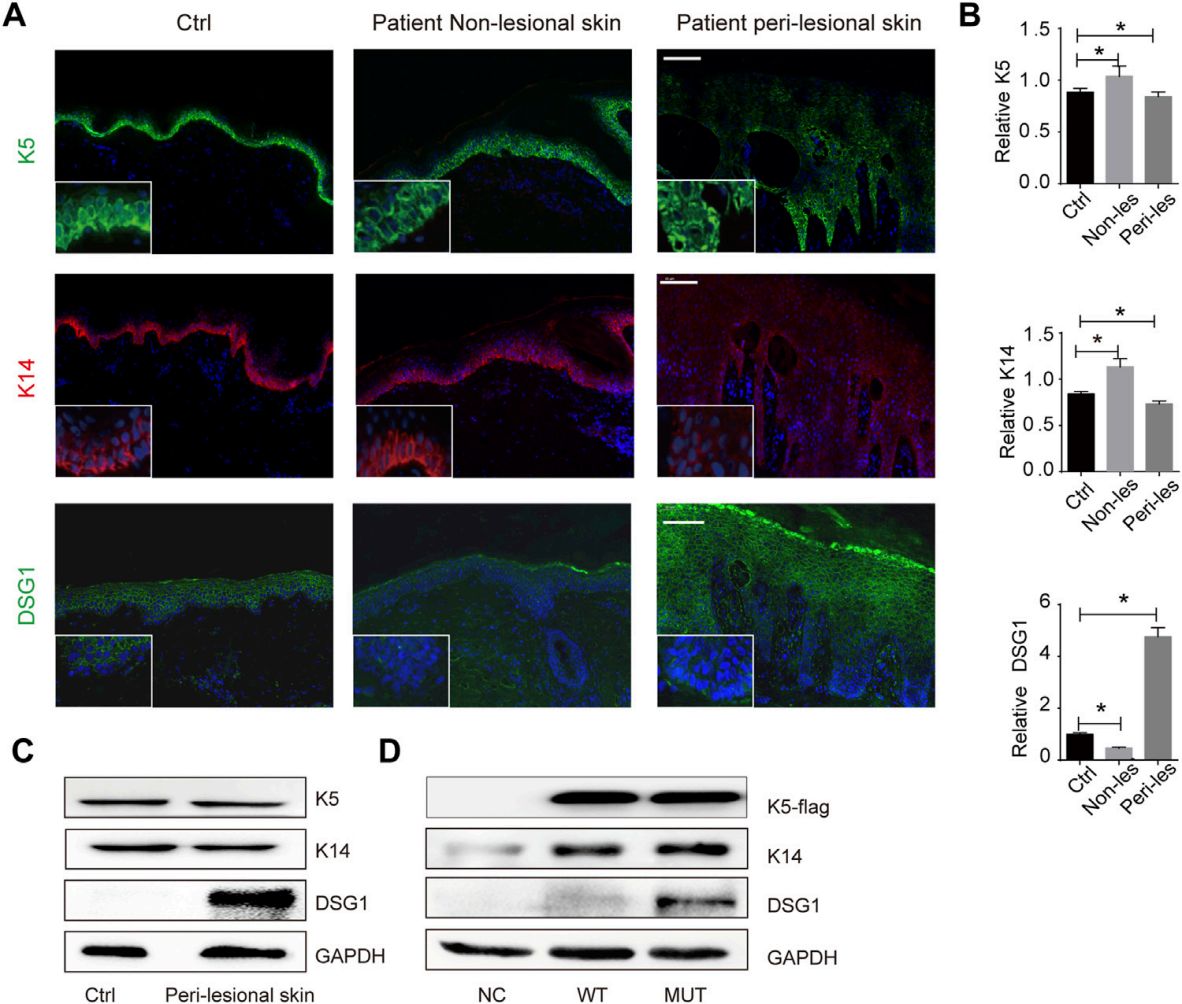
*KRT5* mutation maintains K5/K14 steady protein levels but significantly increased DSG1 level. **(A)** Immunofluorescence staining for K5, K14, and DSG1 revealed slightly decreased expression of K5 and K14 and significantly increased DSG1 in patient peri-lesional skin biopsy. DAPI, 4′,6-diamidino-2-phenylindole. Bars, 100 µm. **(B)** Quantification of the signal in **(A)**. Statistical significance was assessed by ordinary one-way ANOVA, followed by Bonferroni’s multiple comparison. *p < 0.05, Ctrl, *n* = 3. **(C)** Immunoblotting for K5, K14, and DSG1 *in vivo*. **(D)** Immunoblotting for K5-flag, K14, and DSG1 *in vitro*. GAPDH used as a loading control. Three biological replicates were conducted **(C,D)**.

There was a change in K5 expression (a 23% increase in patient non-lesional skin and 2% decrease in patient peri-lesional skin) and K14 expression (a 29% increase in patient non-lesional skin and 14% decrease in patient peri-lesional skin) compared to control, indicating that *KRT5* mutation does not influence the steady levels of K5 and K14 (Figures 2A–C). We then successfully overexpressed wild-type or mutant (c.1474T > C) *KRT5* construct in HaCaT cells, and there were no obvious changes in the steady levels of mutant K5 compared to wild-type K5 (Figure 2D).

**FIGURE 3:**
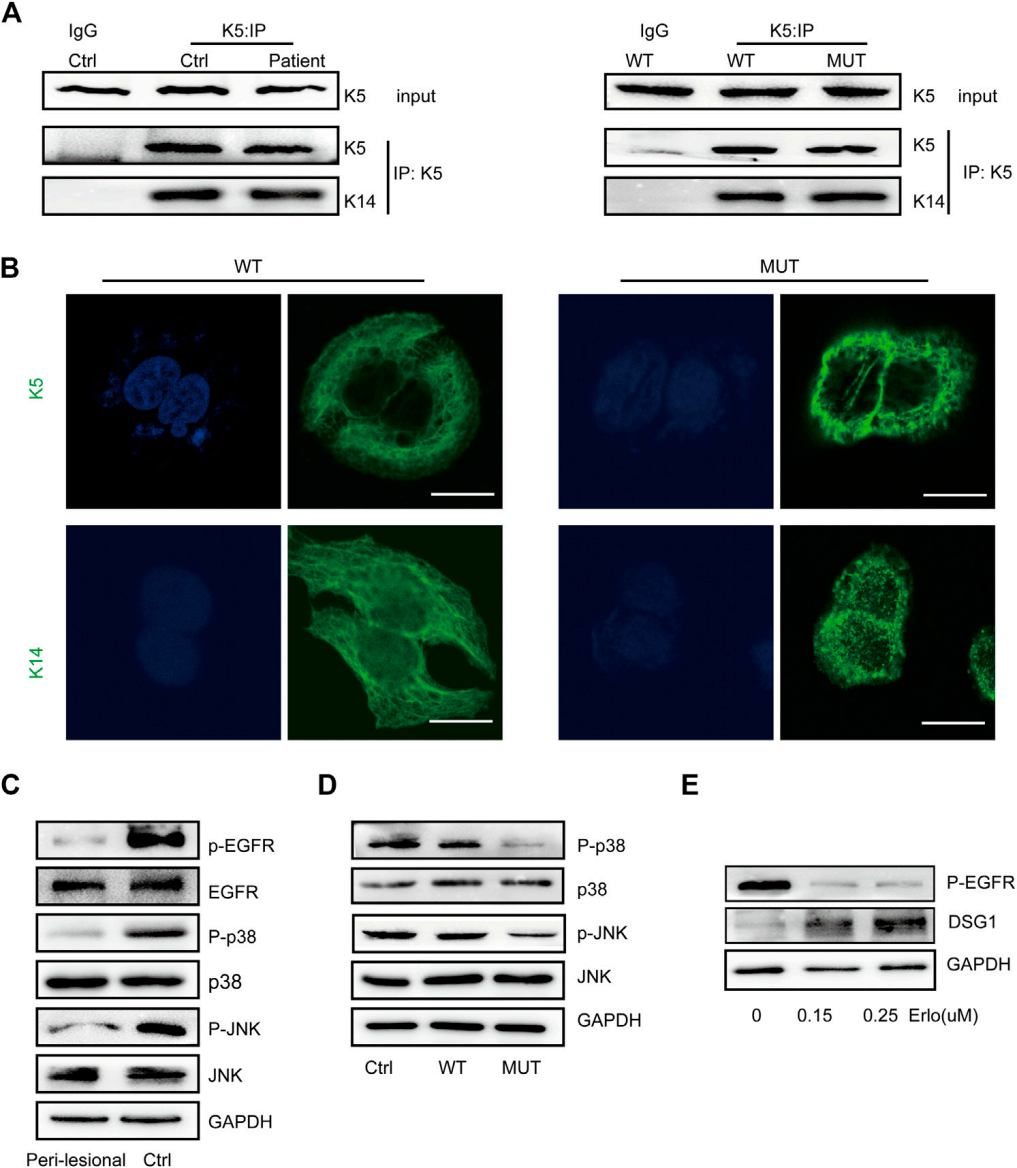
K5 maintained keratin cytoskeleton and DSG1 *via* activating MAPK signaling. **(A)** K14 was co-precipitated with wild-type and mutant K5 in both *in vivo* and *in vitro* models. **(B)** K5 and K14 formed an intact keratin cytoskeleton in wild-type HaCaT cells, but the mutant K5 and K14 proteins aggregated around the nucleus. Bars, 10 µm. **(C,D)** A significant decrease in MAPK signaling was observed in *in vivo* and *in vitro* models. **(E)** Immunoblot analysis showed that EGFR dephosphorylation induced the upregulation of DSG1 expression levels in a concentration-dependent manner. GAPDH, loading control. Three biological replicates were conducted **(A,C,D,E)**.

We performed co-immunoprecipitation (Co-IP) analysis to validate the K5–K14 protein interaction, which indicated that this protein interaction was maintained upon *KRT5* mutation (Figure 3A). Immunostaining for K5 and K14 showed that the keratin network was disturbed, with the irregular clumping of filaments around the nucleus. The percentages of keratin clumped cells transfected with wild-type KRT5 and mutant KRT5 were 7% and 50%, respectively (Figure 3B).

### K5 Maintains DSG1 *via* Activating MAPK Signaling

One hundred eighty-six percent increased desmosomal protein desmoglein 1 (DSG1) in patient peri-lesional skin was observed and increased DSG1 in *in vitro* models was also observed; desmosomal protein desmoglein 3 (DSG3) and desmoplakin (DSP) were not obviously affected (Figures 1F, 2E; Supplementary Figure S2).

To further study the consequences of this mutation, we examined MAPK signaling responses and showed decreased MAPK signaling upon *KRT5* mutation. Downregulated MAPK signaling may contribute to keratin network collapse and abnormalities in desmosomes, consistent with the presence of enlarged intercellular spaces and skin fragility in the patient (Figures 3C,D). As shown in Figure 3E treatment with erlotinib, HCl upregulated the protein level of DSG1.

The experiments in this study indicated that K5 is involved in maintaining the desmosomal cadherin DSG1 and activating MAPK signaling, as DSG1 was upregulated upon EGFR inhibition.

## Discussion

This study strengthens the importance of keratins in the resistance of the epithelia and the maintenance of mechanical stability (Lessard and Coulombe 2012).

EBS was proposed to be a protein misfolding disorder; however, the mechanism of EBS requires further exploration (Herrmann et al., 2002; Chamcheu et al., 2009). In this study, *KRT5* mutation slightly decreased the expression levels of K5 and K14 and mutant K5 could still bind K14 but induce keratin network collapse, suggesting that mutant K5 hampered the proper assembly of keratin IFs and further contribute to decreased resistance to mechanical stress.

Emerging studies have indicated that the desmosomal cadherin regulates keratinocyte morphology, associated with abnormalities in the cytoskeletal architecture, as cells transit through the epidermal layers (Liovic et al., 2009; Homberg et al., 2015). Moreover, impaired desmosomes and downregulation of desmosomal proteins in heterozygous cells from severe dominant EBS patients have been reported (Liovic et al., 2009; Homberg et al., 2015); however, our data showed a significant increase in desmosomes and desmosomal protein in this localized recessive EBS. This suggests that keratins are required to maintain desmosomes, independent of their attachment to the desmosomes (Simpson et al., 2010). The increase in desmosomes observed in this study is different from previous studies; this may be a compensatory response against mechanical stress in localized recessive EBS. Interestingly, we also observed dramatically increased desmosomal protein DSG1 not DSG3 and DSP upon *KRT5* mutation. DSG1 mainly expressed in the upper epidermis while K5 mainly expressed in the basal layer. *KRT5* mutation altered the expression of DSG1, suggesting the regulatory role of this protein, thereby establishing that K5 contributes to the etiology of skin fragility directly or indirectly *via* DSG1.

Overwhelming evidence demonstrates that p38 plays a prominent role in regulating the keratin network and epithelial plasticity (Efimova et al., 2003; Chamcheu et al., 2011). We observed an inhibitory effect on MAPK signaling through EGFR in this localized recessive EBS, while Lane et al. reported a constitutively active ERK pathway and MAPK signaling pathway and a resistance to apoptosis in severe dominant EBS (Wöll et al., 2007; Russell et al., 2010). Severe dominant EBS may compensate for significantly damaged keratin filaments to resist mechanical stress *via* activating MAPK signaling and inhibiting apoptosis, while in mild localized EBS, the function of keratin filament was only partially decreased and can resist mechanical stress to some extent.

Emerging studies have indicated that the DSG1 also plays a role in altering cytoskeletal architecture and regulation of signaling events that coordinate differentiation and stratification (Thomason et al., 2010; Nekrasova et al., 2018). This suggests that downregulated MAPK signaling failed to contribute to the formation of a normal intact cytoskeleton, which in turn decreased the flexibility of the cytoskeleton, compensatively increasing desmosome levels to increase resistance to mechanical stress. We speculate that *KRT5* mutation causes DSG1 misregulation *via* inhibition of EGFR in order to compensate for damaged keratin filaments to resist mechanical stress.

Here, we report that with a *KRT5* mutation, DSG1 was upregulated and accumulated in the cytosol in an EGFR-dependent manner, generating susceptibility to mechanical stress. Our findings identify a hitherto unknown mechanism by which K5 controls intercellular adhesion with potential implications for keratinopathies, which are conditions in which diminished cell adhesion facilitates tissue fragility.

## Data Availability

The raw data supporting the conclusions of this article will be made available by the authors, without undue reservation.
