# Supplementary material for: Damaged Keratin Filament Network Caused by KRT5 Mutations in Localized Recessive Epidermolysis Bullosa Simplex
Source: Front Genet. 2021 Nov 29;12:736610. doi: 10.3389/fgene.2021.736610 (PMC8667171; doi:10.3389/fgene.2021.736610)
Supplement: Supplementary file 1 [file DataSheet3.docx]

# Homo sapiens keratin 5 (KRT5), mRNA

NCBI Reference Sequence: NM_000424.3

[FASTA](https://www.ncbi.nlm.nih.gov/nuccore/NM_000424.3?report=fasta) [Graphics](https://www.ncbi.nlm.nih.gov/nuccore/NM_000424.3?report=graph)

[Go to:](https://www.ncbi.nlm.nih.gov/nuccore/119395753#goto119395753_0)

LOCUS NM_000424 2320 bp mRNA linear PRI 06-FEB-2018

DEFINITION Homo sapiens keratin 5 (KRT5), mRNA.

ACCESSION NM_000424

VERSION NM_000424.3

KEYWORDS RefSeq.

SOURCE Homo sapiens (human)

ORGANISM [Homo sapiens](https://www.ncbi.nlm.nih.gov/Taxonomy/Browser/wwwtax.cgi?id=9606)

tcgacagctctctcgcccagcccagttctggaagggataaaaagggggcatcaccgttcctgggtaacagagccaccttctgcgtcctgctgagctctgttctctccagcacctcccaacccactagtgcctggttctcttgctccaccaggaacaagccaccATGTCTCGCCAGTCAAGTGTGTCCTTCCGGAGCGGGGGCAGTCGTAGCTTCAGCACCGCCTCTGCCATCACCCCGTCTGTCTCCCGCACCAGCTTCACCTCCGTGTCCCGGTCCGGGGGTGGCGGTGGTGGTGGCTTCGGCAGGGTCAGCCTTGCGGGTGCTTGTGGAGTGGGTGGCTATGGCAGCCGGAGCCTCTACAACCTGGGGGGCTCCAAGAGGATATCCATCAGCACTAGTGGTGGCAGCTTCAGGAACCGGTTTGGTGCTGGTGCTGGAGGCGGCTATGGCTTTGGAGGTGGTGCCGGTAGTGGATTTGGTTTCGGCGGTGGAGCTGGTGGTGGCTTTGGGCTCGGTGGCGGAGCTGGCTTTGGAGGTGGCTTCGGTGGCCCTGGCTTTCCTGTCTGCCCTCCTGGAGGTATCCAAGAGGTCACTGTCAACCAGAGTCTCCTGACTCCCCTCAACCTGCAAATCGACCCCAGCATCCAGAGGGTGAGGACCGAGGAGCGCGAGCAGATCAAGACCCTCAACAATAAGTTTGCCTCCTTCATCGACAAGGTGCGGTTCCTGGAGCAGCAGAACAAGGTTCTGGACACCAAGTGGACCCTGCTGCAGGAGCAGGGCACCAAGACTGTGAGGCAGAACCTGGAGCCGTTGTTCGAGCAGTACATCAACAACCTCAGGAGGCAGCTGGACAGCATCGTGGGGGAACGGGGCCGCCTGGACTCAGAGCTGAGAAACATGCAGGACCTGGTGGAAGACTTCAAGAACAAGTATGAGGATGAAATCAACAAGCGTACCACTGCTGAGAATGAGTTTGTGATGCTGAAGAAGGATGTAGATGCTGCCTACATGAACAAGGTGGAGCTGGAGGCCAAGGTTGATGCACTGATGGATGAGATTAACTTCATGAAGATGTTCTTTGATGCGGAGCTGTCCCAGATGCAGACGCATGTCTCTGACACCTCAGTGGTCCTCTCCATGGACAACAACCGCAACCTGGACCTGGATAGCATCATCGCTGAGGTCAAGGCCCAGTATGAGGAGATTGCCAACCGCAGCCGGACAGAAGCCGAGTCCTGGTATCAGACCAAGTATGAGGAGCTGCAGCAGACAGCTGGCCGGCATGGCGATGACCTCCGCAACACCAAGCATGAGATCTCTGAGATGAACCGGATGATCCAGAGGCTGAGAGCCGAGATTGACAATGTCAAGAAACAGTGCGCCAATCTGCAGAACGCCATTGCGGATGCCGAGCAGCGTGGGGAGCTGGCCCTCAAGGATGCCAGGAACAAGCTGGCCGAGCTGGAGGAGGCCCTGCAGAAGGCCAAGCAGGACATGGCCCGGCTGCTGCGTGAGTACCAGGAGCTCATGACACCAAGCTGGCCCTGGACGTGGAGATCGCCACTTACCGCAAGCTGCTGGAGGGCGAGGAATGCAGACTCAGTGGAGAAGGAGTTGGACCAGTCAACATCTCTGTTGTCACAAGCAGTGTTTCCTCTGGATATGGCAGTGGCAGTGGCTATGGCGGTGGCCTCGGTGGAGGTCTTGGCGGCGGCCTCGGTGGAGGTCTTGCCGGAGGTAGCAGTGGAAGCTACTACTCCAGCAGCAGTGGGGGTGTCGGCCTAGGTGGTGGGCTCAGTGTGGGGGGCTCTGGCTTCAGTGCAAGCAGTGGCCGAGGGCTGGGGGTGGGCTTTGGCAGTGGCGGGGGTAGCAGCTCCAGCGTCAAATTTGTCTCCACCACCTCCTCCTCCCGGAAGAGCTTCAAGAGCTAAgaacctgctgcaagtcactgccttccaagtgcagcaacccagcccatggagattgcctcttctaggcagttgctcaagccatgttttatccttttctggagagtagtctagaccaagccaattgcagaaccacattctttggttcccaggagagccccattcccagcccctggtctcccgtgccgcagttctatattctgcttcaaatcagccttcaggtttcccacagcatggcccctgctgacacgagaacccaaagttttcccaaatctaaatcatcaaaacagaatccccaccccaatcccaaattttgttttggttctaactacctccagaatgtgttcaataaaatgcttttataatataaaaaaaaaaaaaaaaaaa

## KRT5c-E05-9F/R

|  | **Sequence (5'->3')** | **Template strand** | **Length** | **Start** | **Stop** | **Tm** | **GC%** | **Self complementarity** | **Self 3' complementarity** |
| --- | --- | --- | --- | --- | --- | --- | --- | --- | --- |
| **Forward primer** | AGTATGAGGAGATTGCCAAC | Plus | 20 | 77 | 96 | 54.82 | 45.00 | 5.00 | 3.00 |
| **Reverse primer** | AGTAGTAGCTTCCACTGCTA | Minus | 20 | 642 | 623 | 54.99 | 45.00 | 8.00 | 3.00 |
| **Product length** | 566 | | | | | | | | |

**Products on potentially unintended templates**

>[NM_000424.3](https://www.ncbi.nlm.nih.gov/entrez/viewer.fcgi?db=nucleotide&id=119395753) Homo sapiens keratin 5 (KRT5), mRNA

product length = 567

Forward primer 1 AGTATGAGGAGATTGCCAAC 20

Template 1197 .................... 1216

Reverse primer 1 AGTAGTAGCTTCCACTGCTA 20

Template 1763 .................... 1744

GACACCTCAGTGGTCCTCTCCATGGACAACAACCGCAACCTGGACCTGGATAGCATCATCGCTGAGGTCAAGGCCCAGTATGAGGAGATTGCCAACCGCAGCCGGACAGAAGCCGAGTCCTGGTATCAGACCAAGTATGAGGAGCTGCAGCAGACAGCTGGCCGGCATGGCGATGACCTCCGCAACACCAAGCATGAGATCTCTGAGATGAACCGGATGATCCAGAGGCTGAGAGCCGAGATTGACAATGTCAAGAAACAGTGCGCCAATCTGCAGAACGCCATTGCGGATGCCGAGCAGCGTGGGGAGCTGGCCCTCAAGGATGCCAGGAACAAGCTGGCCGAGCTGGAGGAGGCCCTGCAGAAGGCCAAGCAGGACATGGCCCGGCTGCTGCGTGAGTACCAGGAGCTCATGACACCAAGCTGGCCCTGGACGTGGAGATCGCCACTTACCGCAAGCTGCTGGAGGGCGAGGAATGCAGACTCAGTGGAGAAGGAGTTGGACCAGTCAACATCTCTGTTGTCACAAGCAGTGTTTCCTCTGGATATGGCAGTGGCAGTGGCTATGGCGGTGGCCTCGGTGGAGGTCTTGGCGGCGGCCTCGGTGGAGGTCTTGCCGGAGGTAGCAGTGGAAGCTACTACTCCAGCAGCAGTGGGGGTGTCGGCCTAGGTGGTGGGCTCAGTGTGGGGGGCTCTGGCTTCAGTGCAAGCAGTGGCCGAGGGCTGGGGGTGGGCTTTGGCAGTGGCGGGGGTAGCAGCTCCAGCGTCAAATTTGTCTCCACCACCTCCTCCTCCCGGAAGAGCTTCAAGAGCTAAgaacctgctgcaagtcactgccttccaagtgcagcaacccagcccatggagattgcctcttctaggcagttgctcaagccatgttttatccttttctggagagtagtctagaccaagccaattgcagaaccacattctttggttcccaggagagccccattcccagcccctggtctcccgtgccgcagttctatattctgcttcaaatcagccttcaggtttccc
