# Supplementary material for: Damaged Keratin Filament Network Caused by KRT5 Mutations in Localized Recessive Epidermolysis Bullosa Simplex
Source: Front Genet. 2021 Nov 29;12:736610. doi: 10.3389/fgene.2021.736610 (PMC8667171; doi:10.3389/fgene.2021.736610)
Supplement: Supplementary file 2 [file DataSheet2.docx]

Supplementary Materials for

**Damaged Keratin Filament Network Caused by *KRT5* Mutations in Localized Recessive Epidermolysis Bullosa Simplex**

*Fuying Chen^1,2^, Lei Yao^3^, Xue Zhang^1,2^, Yan Gu^1,2^, Hong Yu^1^, Zhirong Yao^1,2,*^, Jia Zhang^1,2,*^, and Ming Li ^1,2,^*^*^

**The first two authors contributed equally to this work.**

**# Correspondence:** M. Li, MD, PhD, Department of Dermatology, Xinhua Hospital, Shanghai Jiaotong University School of Medicine, 1665 Kongjiang Road, Shanghai 200092, China. Tel: +86 2125078571, E-mail: [liming01@xinhuamed.com.cn, J](mailto:liming01@xinhuamed.com.cn,%20J). Zhang, MD, PhD, Department of Dermatology, Hospital, Shanghai Jiaotong University School of Medicine, 1665 Kongjiang Road, Shanghai 200092, China, Email: [zhangjia@xinhuamed.com.cn](mailto:zhangjia@xinhuamed.com.cn), or Z.R. Yao, MD, PhD, Department of Dermatology, Xinhua Hospital, Shanghai Jiaotong School of Medicine, 1665 Kongjiang Road, Shanghai 200092, China. Tel.: +86 2125078570 Fax: +86 2165030840, E-mail: [yaozhirong@xinhuamed.com.cn](mailto:yaozhirong@xinhuamed.com.cn).

Bioinformatics analysis

To estimate potential effect of Ser492Pro mutation on the function of human KRT5, we integrated multiple bioinformatics tools to predict the structural, functional and energy change. Firstly, two online tools, ConSurf (Ashkenazy, Erez, Martz, Pupko and Ben-Tal 2010) and PolyPhen-2 (Adzhubei et al. 2010), were used to generate the conservation score and the possible impact on structure and function, respectively. To predict the association between this mutation and disease, MutationTaster server (Schwarz, Cooper, Schuelke and Seelow 2014) was used. And then, modeling of wide type and mutate type was conducted by I-TASSER server (Yang et al. 2015) to access the 3D structure of protein. DUET web server (Pires, Ascher and Blundell 2014) was performed to quantitatively compute the free energy change (ΔΔG). The complex structures and energy scores between KRT5 and KRT14 were obtained by PRISM server (Baspinar, Cukuroglu, Nussinov, Keskin and Gursoy 2014). Protein complex with the lowest energy was selected for the followed analysis. At last, PyMOL Molecular Graphics system (Version 1.3, Schrodinger LCC) was used to survey the mutation’s effect on protein structure.

**Supplementary Table.1 A list of antibodies used in this study.**

| KRT5 anti-rabbit | ab52635 |
| --- | --- |
| KRT14 anti-mouse | ab7800 |
| DSG1 anti-mouse | ab12077 |
| DSG3 anti-mouse | ab14416 |
| DSP anti-mouse | ab16434 |
| Phosphor-EGFR anti-rabbit | CST 3777 |
| EGFR anti-rabbit | CST 4267 |
| GAPDH anti-mouse | Thermofisher, 14-9523-82 |
| p38 anti-rabbit | CST 8690 |
| P-p38 anti-rabbit | CST 4511 |
| JNK anti-rabbit | CST 9252 |
| p-JNK anti-rabbit | CST 4668 |
| anti-Mouse Secondary Antibody, Alexa Fluor 568 | Thermofisher, A-11004 |
| anti-Rabbit Secondary Antibody, Alexa Fluor 488 | Thermofisher, A-11008 |
| anti-Mouse IgG Secondary Antibody, Alexa Fluor 488 | Thermofisher, A-21202 |

**Supplementary Figure**

**
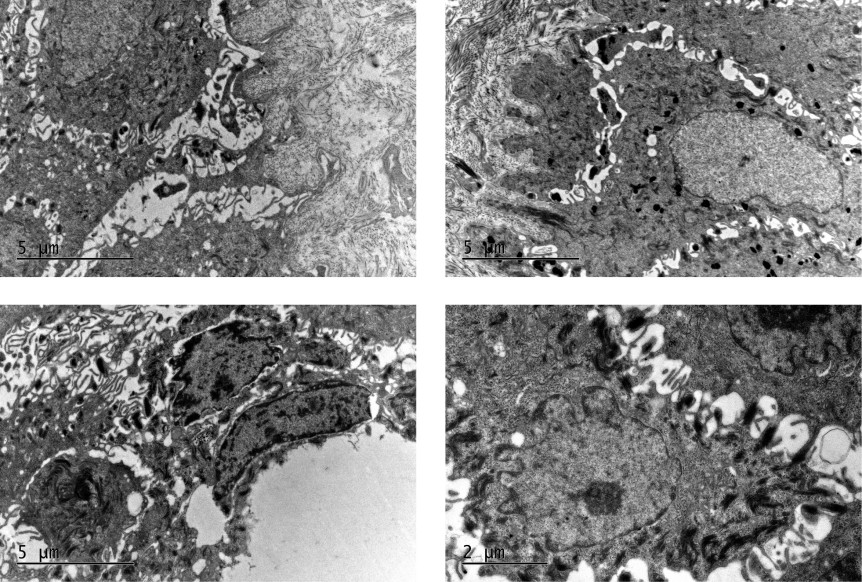
**

**Supplement Figure 1.** Clumped keratin filament and normal keratin filament can be observed in keratinocytes of patient. The intercellular space between the keratinocytes of the patient is larger than that of the normal person, and especially in the intercellular space at the location of the blister.

**
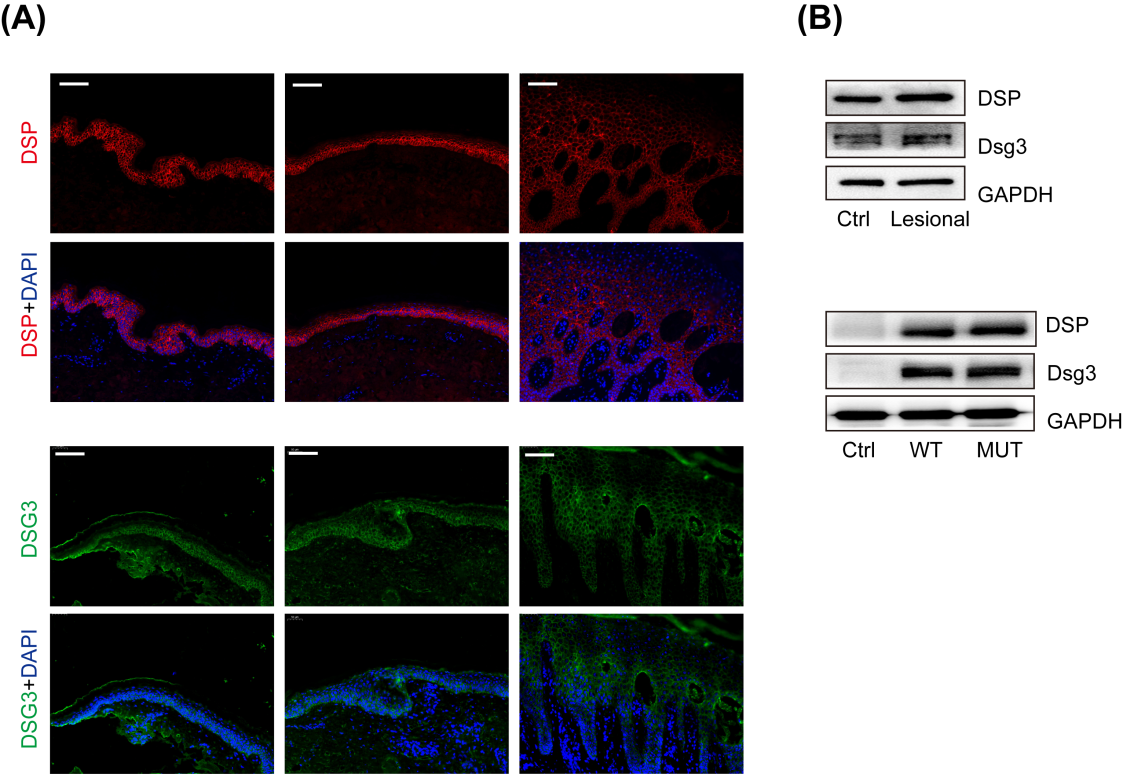
**

**Supplement Figure 2.** Moderately increases in DSG3 and DSP were observed by immunofluorescence and western blotting in patient samples and no obviously changes of DSG3 and DSP between wildtype and mutant KRT5 in HaCaT cells.

**REFERENCES**

Adzhubei, I. A., S. Schmidt, L. Peshkin, V. E. Ramensky, A. Gerasimova, P. Bork, et al.(2010). A method and server for predicting damaging missense mutations. *Nat Methods* 7:248-249.10.1038/nmeth0410-248

Ashkenazy, H., E. Erez, E. Martz, T. Pupko, and N. Ben-Tal.(2010). ConSurf 2010: calculating evolutionary conservation in sequence and structure of proteins and nucleic acids. *Nucleic Acids Res* 38:W529-533.10.1093/nar/gkq399

Baspinar, A., E. Cukuroglu, R. Nussinov, O. Keskin, and A. Gursoy.(2014). PRISM: a web server and repository for prediction of protein-protein interactions and modeling their 3D complexes. *Nucleic Acids Res* 42:W285-289.10.1093/nar/gku397

Pires, D. E., D. B. Ascher, and T. L. Blundell.(2014). DUET: a server for predicting effects of mutations on protein stability using an integrated computational approach. *Nucleic Acids Res* 42:W314-319.10.1093/nar/gku411

Schwarz, J. M., D. N. Cooper, M. Schuelke, and D. Seelow.(2014). MutationTaster2: mutation prediction for the deep-sequencing age. *Nat Methods* 11:361-362.10.1038/nmeth.2890

Yang, J., R. Yan, A. Roy, D. Xu, J. Poisson, and Y. Zhang.(2015). The I-TASSER Suite: protein structure and function prediction. *Nat Methods* 12:7-8.10.1038/nmeth.3213
