# Supplementary material for: Damaged Keratin Filament Network Caused by KRT5 Mutations in Localized Recessive Epidermolysis Bullosa Simplex
Source: Front Genet. 2021 Nov 29;12:736610. doi: 10.3389/fgene.2021.736610 (PMC8667171; doi:10.3389/fgene.2021.736610)
Supplement: Supplementary file 3 [file DataSheet1.docx]

Supplementary Materials for

**Damaged Keratin Filament Network Caused by *KRT5* Mutations in Localized Recessive Epidermolysis Bullosa Simplex**

*Fuying Chen^1,2^, Lei Yao^3^, Xue Zhang^1,2^, Yan Gu^1,2^, Hong Yu^1^, Zhirong Yao^1,2,*^, Jia Zhang^1,2,*^, and Ming Li ^1,2,^*^*^

**The first two authors contributed equally to this work.**

**# Correspondence:** M. Li, MD, PhD, Department of Dermatology, Xinhua Hospital, Shanghai Jiaotong University School of Medicine, 1665 Kongjiang Road, Shanghai 200092, China. Tel: +86 2125078571, E-mail: [liming01@xinhuamed.com.cn, J](mailto:liming01@xinhuamed.com.cn,%20J). Zhang, MD, PhD, Department of Dermatology, Hospital, Shanghai Jiaotong University School of Medicine, 1665 Kongjiang Road, Shanghai 200092, China, Email: [zhangjia@xinhuamed.com.cn](mailto:zhangjia@xinhuamed.com.cn), or Z.R. Yao, MD, PhD, Department of Dermatology, Xinhua Hospital, Shanghai Jiaotong School of Medicine, 1665 Kongjiang Road, Shanghai 200092, China. Tel.: +86 2125078570 Fax: +86 2165030840, E-mail: [yaozhirong@xinhuamed.com.cn](mailto:yaozhirong@xinhuamed.com.cn).

Interface Residues Contacts between KRT5-WT and KRT14

target1_chain_resName_resNo <--> target2_chain_resName_resNo

KRT5-WT_A_ILE_339 <--> KRT14_A_LEU_426

KRT5-WT_A_ILE_393 <--> KRT14_A_VAL_380

KRT5-WT_A_THR_371 <--> KRT14_A_LEU_401

KRT5-WT_A_LEU_396 <--> KRT14_A_GLN_372

KRT5-WT_A_LEU_396 <--> KRT14_A_ILE_373

KRT5-WT_A_MET_389 <--> KRT14_A_GLN_383

KRT5-WT_A_THR_382 <--> KRT14_A_LEU_387

KRT5-WT_A_ALA_357 <--> KRT14_A_LEU_408

KRT5-WT_A_ILE_400 <--> KRT14_A_LEU_370

KRT5-WT_A_ASP_378 <--> KRT14_A_GLN_394

KRT5-WT_A_ILE_349 <--> KRT14_A_TYR_415

KRT5-WT_A_VAL_403 <--> KRT14_A_GLN_369

KRT5-WT_A_ILE_349 <--> KRT14_A_ILE_412

KRT5-WT_A_LEU_396 <--> KRT14_A_MET_376

KRT5-WT_A_VAL_403 <--> KRT14_A_TYR_366

KRT5-WT_A_VAL_342 <--> KRT14_A_LEU_419

KRT5-WT_A_LYS_364 <--> KRT14_A_LEU_401

KRT5-WT_A_LYS_364 <--> KRT14_A_ILE_400

KRT5-WT_A_ARG_397 <--> KRT14_A_ILE_373

KRT5-WT_A_ILE_393 <--> KRT14_A_ILE_377

KRT5-WT_A_ILE_393 <--> KRT14_A_MET_376

KRT5-WT_A_ASP_334 <--> KRT14_A_LEU_426

KRT5-WT_A_LEU_335 <--> KRT14_A_SER_427

KRT5-WT_A_LEU_335 <--> KRT14_A_LEU_426

KRT5-WT_A_MET_392 <--> KRT14_A_MET_376

KRT5-WT_A_TRP_360 <--> KRT14_A_VAL_404

KRT5-WT_A_ILE_400 <--> KRT14_A_ILE_373

KRT5-WT_A_TRP_360 <--> KRT14_A_LEU_401

KRT5-WT_A_LEU_368 <--> KRT14_A_LEU_401

KRT5-WT_A_ILE_386 <--> KRT14_A_LEU_387

KRT5-WT_A_ILE_386 <--> KRT14_A_LEU_384

KRT5-WT_A_LEU_410 <--> KRT14_A_THR_362

KRT5-WT_A_TYR_346 <--> KRT14_A_ARG_416

KRT5-WT_A_VAL_342 <--> KRT14_A_GLU_422

KRT5-WT_A_ILE_338 <--> KRT14_A_LEU_426

KRT5-WT_A_SER_353 <--> KRT14_A_ILE_412

KRT5-WT_A_TYR_346 <--> KRT14_A_LEU_419

KRT5-WT_A_ILE_338 <--> KRT14_A_GLU_422

KRT5-WT_A_GLN_345 <--> KRT14_A_LEU_419

KRT5-WT_A_GLU_356 <--> KRT14_A_LEU_408

Interface Residues Contacts between KRT5-Pro492 and KRT14

target1_chain_resName_resNo <--> target2_chain_resName_resNo

KRT5-Pro492_A_LEU_396 <--> KRT14_A_ILE_412

KRT5-Pro492_A_ASP_320 <--> KRT14_A_GLU_344

KRT5-Pro492_A_MET_392 <--> KRT14_A_LYS_405

KRT5-Pro492_A_MET_389 <--> KRT14_A_LEU_402

KRT5-Pro492_A_TYR_361 <--> KRT14_A_MET_376

KRT5-Pro492_A_ILE_400 <--> KRT14_A_ILE_412

KRT5-Pro492_A_SER_322 <--> KRT14_A_GLN_348

KRT5-Pro492_A_LEU_379 <--> KRT14_A_MET_391

KRT5-Pro492_A_VAL_323 <--> KRT14_A_GLU_344

KRT5-Pro492_A_THR_321 <--> KRT14_A_GLU_344

KRT5-Pro492_A_ASN_332 <--> KRT14_A_MET_351

KRT5-Pro492_A_ILE_386 <--> KRT14_A_TYR_398

KRT5-Pro492_A_SER_359 <--> KRT14_A_VAL_380

KRT5-Pro492_A_SER_322 <--> KRT14_A_GLU_344

KRT5-Pro492_A_VAL_403 <--> KRT14_A_ILE_412

KRT5-Pro492_A_SER_322 <--> KRT14_A_SER_347

KRT5-Pro492_A_ILE_393 <--> KRT14_A_LEU_401

KRT5-Pro492_A_VAL_403 <--> KRT14_A_TYR_415

KRT5-Pro492_A_ILE_393 <--> KRT14_A_LYS_405

KRT5-Pro492_A_LEU_335 <--> KRT14_A_LEU_355

KRT5-Pro492_A_HIS_317 <--> KRT14_A_ASN_340

KRT5-Pro492_A_GLN_313 <--> KRT14_A_ARG_336

KRT5-Pro492_A_SER_359 <--> KRT14_A_ILE_377

KRT5-Pro492_A_SER_359 <--> KRT14_A_MET_376

KRT5-Pro492_A_LEU_368 <--> KRT14_A_VAL_380

KRT5-Pro492_A_SER_359 <--> KRT14_A_ILE_373

KRT5-Pro492_A_LEU_396 <--> KRT14_A_LYS_405

KRT5-Pro492_A_LEU_368 <--> KRT14_A_LEU_384

KRT5-Pro492_A_LEU_396 <--> KRT14_A_GLU_409

KRT5-Pro492_A_HIS_375 <--> KRT14_A_MET_391

KRT5-Pro492_A_GLU_358 <--> KRT14_A_ILE_377

KRT5-Pro492_A_LYS_404 <--> KRT14_A_ILE_412

KRT5-Pro492_A_LYS_404 <--> KRT14_A_TYR_415

KRT5-Pro492_A_ILE_400 <--> KRT14_A_LEU_408

KRT5-Pro492_A_VAL_342 <--> KRT14_A_TYR_366

KRT5-Pro492_A_CYS_407 <--> KRT14_A_TYR_415

KRT5-Pro492_A_MET_389 <--> KRT14_A_LYS_405

KRT5-Pro492_A_TRP_360 <--> KRT14_A_VAL_380

KRT5-Pro492_A_TRP_360 <--> KRT14_A_LEU_384

KRT5-Pro492_A_LEU_410 <--> KRT14_A_LEU_419

KRT5-Pro492_A_GLN_406 <--> KRT14_A_LEU_419

KRT5-Pro492_A_TYR_346 <--> KRT14_A_GLN_369
